# Experience of treating congenital complete atrioventricular block with epicardial pacemaker in infants and young children: a retrospective study

**DOI:** 10.1186/s12872-023-03620-1

**Published:** 2023-11-21

**Authors:** Linhong Song, Qiang Meng, Changgen Liu, Gang Wang, Hui Wang, Gengxu Zhou, Zhichun Feng

**Affiliations:** 1 Department of Pediatric Cardiology, Faculty of Pediatrics, The Seventh Medical Center of Chinese PLA General Hospital, NO. 5 Nanmencang, Dongcheng District, Beijing, 100700 China; 2 Second School of Clinical Medicine, Southern Medical University, Guangzhou, China

**Keywords:** Congenital complete heart block, Pacemaker implantation, Pacemaker, Neonates and infants

## Abstract

**Background:**

This article summarizes the treatment experience for congenital complete atrioventricular block (CCAVB) in newborns and infants, and discusses the necessity and feasibility of treating CCAVB with permanent pacemaker implantation in this population.

**Methods:**

In this study, the clinical data and follow-up results of nine children admitted at our center with CCAVB from January 2005 to March 2023 were retrospectively analyzed. Among them, two children received early implantation of permanent pacemakers (within 1 year of age), two children received non-early implantation (1 year or older), and the remaining five children received no pacemaker implantation. CCAVB diagnosis was confirmed by clinical symptoms and clinical examinations, including electrocardiography and echocardiography before surgery. After surgery, the pacing and sensing functions of the pacemaker were observed using electrocardiography, echocardiography, and pacing threshold monitoring. A comprehensive assessment of the treatment efficacy was conducted, encompassing improvements in clinical symptoms, growth and development, as well as the absence of any additional potential complications. The children who did not receive pacemaker implantation were followed up.

**Results:**

Among the four children who successfully received pacemaker implantation, one child who received non-early implantation died. For the remaining three children, the threshold level, amplitude, impedance, and minute ventilation sensor function of the pacemaker were good during the follow-up period, with a heart rate at the pacing rate. The growth and development of the aforementioned patients who received pacemaker implantation demonstrated adherence to the percentile curve, and their motor and cognitive development remained unaffected. However, among the children who did not undergo pacemaker implantation, two experienced death, while three were lost to follow-up, thereby limiting the evaluation of their long-term outcomes.

**Conclusions:**

Early implantation of an epicardial pacemaker at an early stage in newborns and infants diagnosed with CCAVB can significantly improve clinical symptoms without affecting their growth and development. These data are in line with current literature and suggest that early implantation of an epicardial pacemaker in newborns and infants diagnosed with CCAVB but further studies are needed.

## Introduction

Congenital complete atrioventricular block (CCAVB) is a rare congenital heart disease [1] occurring in the approximately 1 in 15,000 to 20,000 live births worldwide [2, 3]. The fetal atrioventricular block is most commonly associated with congenital abnormalities or the presence of maternal autoantibodies [3–7]. However, complete heart block (CHB), comprising congenital and acquired forms, is a comparatively infrequent condition that can manifest following viral infections, as a consequence of infections or drug therapies, or may even be diagnosed without any identifiable cause. The criteria for CCAVB diagnosis include the presence of conduction abnormalities prenatally or within the first 27 days of life [2, 8–10]. Given that untreated CCAVB is associated with high the mortality [11, 12] permanent pacemaker implantation is necessary for infants with CCAVB [3, 4]. In the last decade, implanting pacemakers in newborns and infants was not a common practice. This was mainly because of physiological limitations, including the small size of the patients, and constraints in medical expertise and healthcare resources [13, 14]. The Medtronic pacemaker system (Medtronic, Singapore) is one of the smallest pacemaker systems available in the market, which has been demonstrated to be safe and efficacious in large-scale use in adults [15]. Currently, there is limited clinical data and experience of permanent pacemaker implantation in newborns and infants with CCAVB. This study aimed to quantify the complications of implantation and outcome at our center. A retrospective analysis of nine cases of CCAVB was performed to evaluate their diagnosis, treatment, and follow-up, including four infants who received permanent Medtronic pacemaker (RelieaREDR01/RelieaADSR01/SensiaSED01) implantation. Our study provides relevant experience for future clinical use in permanent pacemaker therapy for newborns and infants with CCAVB.

## Materials and methods

In this study, nine cases of newborns and infants diagnosed with CCAVB from January 2005 to March 2023 were enrolled. The inclusion criteria were as follows: (1) electrocardiogram or 24-hour dynamic electrocardiogram indicating the presence of third-degree atrioventricular block and (2) clear diagnosis of complete congenital heart block during the neonatal and infant period. Patients were excluded based on the presence of acquired factors such as hypertrophic cardiomyopathy, various types of muscular dystrophy, viral myocarditis, rheumatic fever, infection, acquired long Q-T interval syndrome (LQTS), familial dysautonomia, post-cardiac surgery, radiofrequency ablation, high vagal tone, electrolyte disturbances and drug effects. The study included 4 males and 5 females, aged between 15 min and 485 days (1 year and 4 months) and diagnosed with CCAVB based on a complete prenatal or postnatal electrocardiogram. The indications for pacemaker implantation were selected in line with the American College of Cardiology/American Heart Association guidelines for pacemaker implantation in children and adolescents [16, 17]. We observed that four pediatric patients who underwent pacemaker implantation exhibited heart rates ranging from 40 to 60 beats per minute, and hence met the indications specified in the guidelines of the American College of Cardiology/American Heart Association [16, 17].

### Research methods

We adopted a the retrospective research method to explore and evaluate the outcomes of nine cases of newborns and infants with CCAVB following permanent pacemaker implantation. Comprehensive data were collected during the surgical procedure and subsequent follow-up examinations for patients who underwent pacemaker implantation. This included detailed information regarding the operation itself, as well as the results observed during the follow-up period. In contrast, for patients who did not receive pacemaker implantation, electrocardiogram data and clinical symptoms were recorded during the follow-up period. For both groups, patient-level clinical data were collected, including gestational age (the earliest record of bradycardia), atrial and ventricular rates, gestational age at birth, maternal medication history during pregnancy, obstetric history, prenatal ultrasound (cardiac) and postnatal cardiac ultrasound results, degree of heart block after birth, mode of delivery (vaginal delivery or cesarean section), Apgar score, birth weight, age and weight at pacemaker implantation, use of mechanical ventilation and positive inotropic agents (isoproterenol) for treatment, and complications related to CCAVB neonatal lupus erythematosus and endocardial fibroelastosis. The Ethics Committee of the Seventh Medical Center of the PLA General Hospital approved this study (2023-34).

### Follow-up and management

Regular follow-up is necessary after the implantation of a permanent pacemaker. Short-term postoperative follow-up was conducted at weeks 1, 8, 16, and 32, then at 6 to 12 months, and annually thereafter. Follow-up included a review of electrocardiograms and echocardiograms, measurement of pacing threshold, observation of pacing and sensing function of the pacemaker, evaluation of pacemaker parameter values, improvement of bradycardia-related clinical manifestations, growth and development status, and assessment of any other complications. Long-term follow-up was performed for 1 to 3 years focusing on the child’s growth, feeding behavior, development status, pacemaker function, parameters, heart rate regulation, and minute ventilation sensor function. In addition, adverse events and tolerability of the pacemaker system were evaluated, and growth and development status were assessed using growth charts from the Chinese Health Organization. Pacemaker parameters such as program mode, minimum rate interval, intrinsic heart rate, impedance, threshold, amplitude, and pulse duration were also recorded during follow-up.

## Results

We retrospectively enrolled nine cases with CCAVB, including five cases that did not receive implantation therapy and four cases that successfully received epicardial pacemaker implantation. Out of the four patients who underwent permanent pacemaker implantation, three exhibited favorable postoperative recovery, characterized by the resolution of clinical symptoms and optimal pacemaker performance with regard to cardiac pacing and sensing functions. The cardiac pacing threshold tended to be stable 3 months after implantation. In addition, no complications such as lead removal, skin necrosis, vascular occlusion, thrombosis, atrioventricular valve insufficiencies, and cardiac malfunction, were observed in all patients. However, one patient’s guardians refused to continue treatment and thus were discharged before being cured. The patient was readmitted at the age of 1 year and 5 months due to severe pneumonia and heart failure. Echocardiography showed that the ductus arteriosus was not closed with a diameter of 5.9 mm. Subsequently, a permanent pacemaker was surgically implanted and ductus arteriosus ligation was performed. After the surgery, the symptoms of pulmonary congestion and bradycardia caused by the non-closure of the ductus arteriosus resolved. However, due to severe pulmonary infection, poor lung function, and pulmonary consolidation, as well as *Acinetobacter baumannii* which was detected in blood culture, the infection was difficult to reverse and led to severe sepsis, septic shock, and multiple organ failure, eventually resulting in death. Among the patients who did not undergo pacemaker implantation, two patients succumbed to mortality, while an additional three patients were lost to follow-up. Despite our continuous attempts to reach out to the respective families through phone calls, we eventually lost, contact due to a change in their phone number. Detailed information regarding the nine patients during the perinatal period can be found in Table 1.

**Table 1:** Perinatal statistics of nine patients

Project	Case 1	Case 2	Case 3	Case 4	Case 5	Case 6	Case 7	Case 8	Case 9	
Sex	M	M	F	F	F	F	F	M	Male	
GA (wks + d)	36^+ 1^	32	39^+ 2^	39	36^+ 4^	38	38	39	40	
BW (kg)	2.53	1.57	3.3	3	2.42	3	2.84	3.1	3.9	
Delivery method	Cesarean	Cesarean	Section	Section	Cesarean	Cesarean	Cesarean	Cesarean	Section	
APGAR	9/9/9	Unknown	Unknown	9/9/9	9/9/9	9/9/10	8/9/9	5/8/9	9/10/10	
Maternal SS-A/Ro positive/SS-B/La positive	Y	N	N	N	Y	N	N	N	N	
GA at Fetal HRDeceleration(wks)	20	Unknown	17	Unknown	Unknown	Unknown	Unknown	Unknown	Unknown	
Fetal ultrasound	third-degree atrioventricular block); mild regurgitation of mitral and tricuspid valves and pulmonary artery valve; pericardial effusion	N	N	N	N	N	N	N	N	
Amniotic fluid	Normal	Unknown	Normal	Normal	Normal	Normal	Slightly decreased, with abnormal characteristics	Normal	Meconium-stained	
Fetal edema present	Y	Unknown	Unknown	N	Unknown	N	N	N	N	
Fetal HR (bpm)	40–50	Unknown	50–60	Unknown	Unknown	40–50	55–60	Unknown	55–80	
Postnatal HR (bpm)	40–50	40–50	50–60	40–60	40–55	40–55	50–60	45–60	60–84	

Leg: F female, M male, GA gestational age, a year, wks weeks, d days, kg kilogram, bpm beats per minute, HR heart rate, Y yes, N none

Among the nine patients, three received mechanical ventilation support, two received oxygen therapy and three did not receive oxygen therapy. Six patients received isoprenaline hydrochloride injection and other drugs but they did not improve the atrioventricular block. Three patients did not receive drug treatment, and 1 patient in this group was diagnosed with CCAVB at a gestational age of 20 weeks and was treated with prednisone acetate during pregnancy and dexamethasone at a gestational age of 32 weeks. Isoprenaline was administered after birth to increase heart rate, but there was no significant improvement. Six patients received a diagnosis of CCAVB and were treated with isoprenaline, but no significant improvement was observed, In contrast, three patients did not receive any drug treatment. Meanwhile, we observed that the fetal heart rate was lower in fetuses with edema than in those without edema. Two patients’ mothers had systemic lupus erythematosus, but none indicated having connective tissue diseases. A total of four patients underwent pacemaker implantation at the following ages: 2 days, 78 days (2 months and 17 days), 485 days (1 year and 4 months), and 365 days (1 year). The average weight of these patients at the time of implantation was 4.98 kg. The lead was located on the epicardium of the heart and the device was placed in the upper left abdomen, whereas the other five patients did not receive pacemaker implantation. Specific examination and treatment information for the nine patients is shown in Table 2.

**Table 2:** Perinatal medication and examination statistics of nine pediatric patients

Project	Case 1	Case 2	Case 3	Case 4	Case 5	Case 6	Case 7	Case 8	Case 9
Whether to merge endocardial elastosis	N	N	N	N	N	N	N	N	N
Isoproterenol hydrochloride injection	Y	Y	Y	N	Y	Y	N	Y	N
Mechanical ventilation	Y	N	Y	N	N	N	N	Y	N
Oxygen	Y	Y	Y	N	Y	Y	N	Y	N
Echocardiogram	N	Patent foramen ovale; patent ductus arteriosus	Patent ductus arteriosus	Enlarged heart with mild mitral and tricuspid regurgitation	Patent foramen ovale; patent ductus arteriosus	Enlarged heart with patent foramen ovale, patent ductus arteriosus, and pulmonary hypertension	N	N	N
Electrocardiogram	Sinus rhythm, abnormal ECG; third-degree atrioventricular block with junctional escape rhythm Sinus rhythm	Sinus rhythm, abnormal ECG; third-degree atrioventricular block with junctional escape rhythm Sinus rhythm,	Sinus rhythm, abnormal ECG; third-degree atrioventricular block with junctional escape rhythm	Sinus rhythm, abnormal ECG; third-degree atrioventricular block with junctional escape rhythm; complete atrioventricular dissociation	Abnormal ECG; complete atrioventricular dissociation (third-degree atrioventricular block)	Abnormal ECG; third-degree atrioventricular block	Abnormal ECG; third-degree atrioventricular block	Abnormal ECG; third-degree atrioventricular block	Abnormal ECG; third-degree atrioventricular block

Leg: ECG Electrocardiogram, Y yes, N none

The initial programming data of the pacemakers for the four patients are shown in Table 3. There was no adverse event in the four children who received pacemaker implantation during follow-up. The placement of the pacemaker in this study involved positioning it in the mid-lateral wall of the ventricle. Importantly, no atrioventricular dyssynchrony or cardiac dysfunction were observed following pacemaker implantation throughout the follow-up period. Among these patients, pacemakers were programmed in Dual-Chamber Demand Pacing (DDD) mode for three patients, with lower rate intervals set at 75 beats/minute (800 milliseconds), 70 beats/minute (857 milliseconds), and 75 beats/minute (800 milliseconds). The pacemaker of one case was programmed in ventricular inhibited or demand type pacing (VVI) mode, with a lower rate interval of 95 beats/minute (630 milliseconds).

**Table 3:** Pacemaker data for four pediatric patients with implanted pacemakers

Project	Case 1	Case 2	Case 3	Case 4
Age at Implant (d)	2	78	485	365
Weight at Implant (kg)	2.53	3	8.4	9
Initial programming				
Model	RelieaREDR01	RelieaADSR01	SensiaSED01	RelieaREDR01
Mode	DDD	VVI	DDD	DDD
Lower rate	75	95	70	75
Upper tracking rate	160	160	140	140
Intrinsic rhythm (bpm)	45	47	60	62
Polarity	Bipolar	unipolar	Bipolar	Bipolar
V(A)impedance (Ω)	401(357)	389	290(266)	510(480)
R/P -wave(mV)	7.5/6.5	11.2	10.5/4.8	7.5/4.2
V Threshold (V/ms)	0.9/0.4	0.75/0.48	0.6/0.4	0.625/0.4
A Threshold (V/ms)	1.1/0.4	N	1.0/0.4	1.1/0.4
PM size(Length/ width/height, mm)	44.7/47.9/7.5	40.2/42.9/7.5	44.7/47.9/7.5	44.7/47.9/7.5

Leg: wks weeks, d days, kg kilogram, bpm beats per minute, Ω Ohm, mV millivolt, V /ms Volt/milliseconds, pp postpartum, V Ventricular, A Atrial, N none, DDD Dual-Chamber Demand Pacing, VVI Ventricular Inhibited Pacing or Ventricular Demanded Pacing. PM pacemaker

**Fig. 1:**
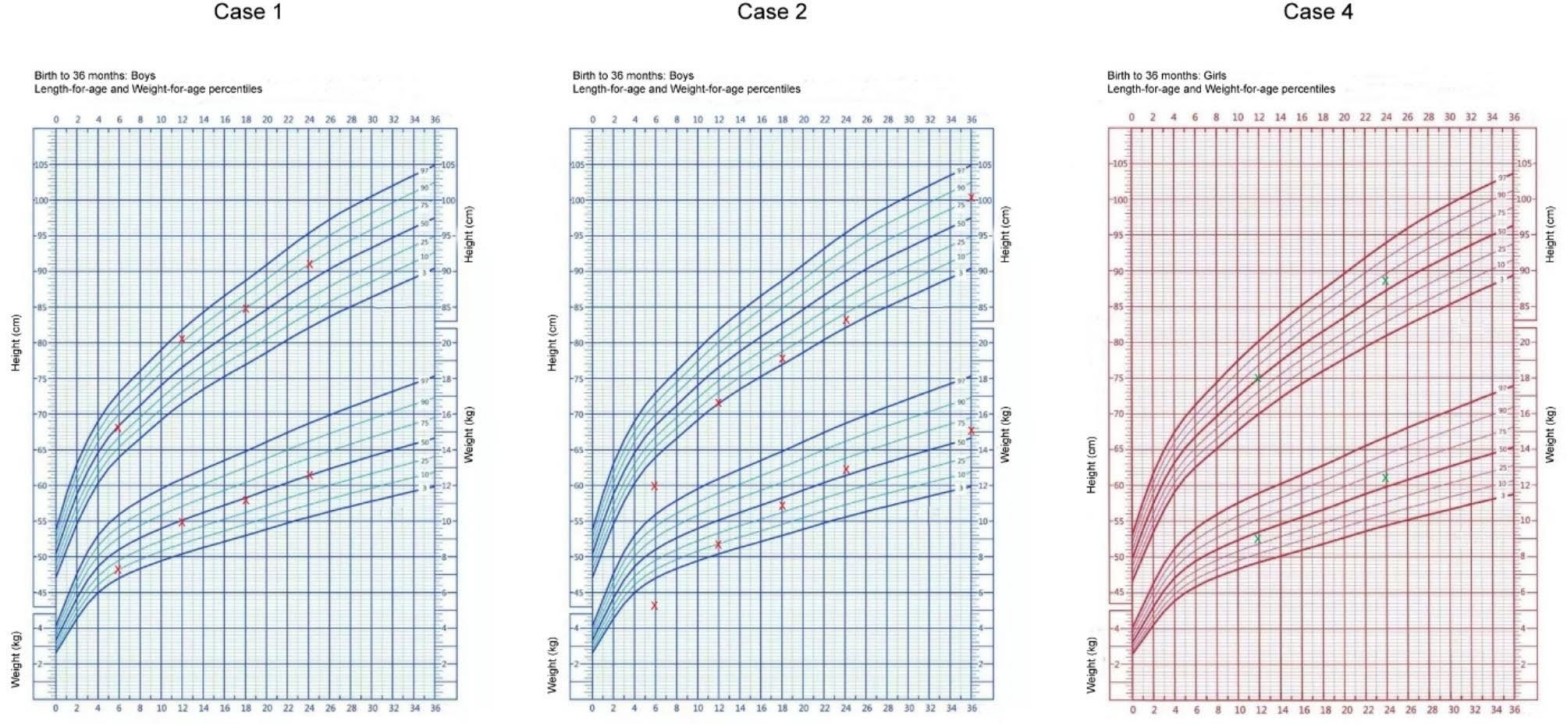
The growth percentile curve of patients 1#, 2#, 4#

Among the four pediatric patients who received pacemaker implantation, three surviving patients were followed up for up to 3 years. Throughou the follow-up period, the pacemaker demonstrated proper functionality in terms of threshold level, amplitude, impedance, and minute ventilation sensor. Patients showed significant improvement in weight and height and catch-up growth along the percentile curve during follow-up (Fig. 1).

The x-axis displays age in months, and the y-axis displays weight in kilograms and height in centimeters on the bottom right and top left, respectively.

## Discussion

In this study, a retrospective analysis was conducted on clinical data obtained from a cohort of nine pediatric patients. We found that permanent pacemaker implantation was effective and safe, which is consistent with the findings from previous investigations [18, 19]. Four of the nine infants with CCAVB received pacemaker therapy and achieved good outcomes during follow-up. For the remaining five patients, their families declined pacemaker implantation due to personal reasons and some were lost to follow-up. CCAVB is relatively rare in infants, and despite its high mortality rate, there is limited clinical data available for newborns and young infants. Studies have reported a mortality rate of as high as 16% in infants, compared with 4-8% for older children. When combined with other congenital heart defects, the mortality rate for both infants and older children rises. Permanent pacemaker implantation is the only effective method [18, 20–22] to prevent sudden death in patients diagnosed with CCAVB [23].

The common treatment methods for CCAVB are medical therapy and pacemaker implantation. Medical therapy involves the use of β-adrenergic agonists such as isoproterenol, dopamine, dobutamine, and adrenaline to improve atrioventricular conduction and increase heart rate [22]. Scholars hold varying opinions among scholars regarding the indications for pacemaker implantation. Some argue scholars believe that for asymptomatic children with CCAVB may postpone pacemaker implantation, deeming it necessary only when symptoms like syncope arise [18]. Other researchers suggest early intervention is particularly necessary for CCAVB diagnosed before birth and permanent pacemakers should be implanted in CCAVB children in early infancy [20]. According to the “Guidelines for Pacemaker Implantation in Children and Adolescents” released by the American College of Cardiology/American Heart Association in 2021, newborns or infants with CCAVB and complex congenital heart disease should be considered for pacemaker implantation when bradycardia and hemodynamic impairment are associated or when the mean ventricular rate is < 60–70 beats/min [16]. In this study, six pediatric patients were treated with isoproterenol hydrochloride. However, there was only minimal improvement observed in their symptoms despite the intervention. Additionally, the study involved nine pediatric cases that met the clinical criteria for pacemaker implantation [16]. Out of these, four cases were implanted with permanent pacemakers and achieved satisfactory results. All patients exhibited an absence of previous clinical symptoms after the procedure, with three patients notably demonstrating positive growth and development. While the manifestations of CCAVB were eliminated in one patient, experienced mortality due to factors not associated with the pacemaker implantation. This incident suggests minimal inherent safety concerns with the pacemaker therapy, as the causality was unrelated to the surgical intervention itself.

The criteria for selection of pacemakers vary notably between infants and adults, reflecting the physiological and anatomical differences across these age groups. Typically, endocardial implantation is the preferred choice for older children and adults. Nonetheless, in the case of neonates and infants weighing under 10–15 kg, as well as individuals with vascular or cardiac anomalies posing challenges for venous implantation, serious consideration should be given to epicardial implantation [24]. Although several studies have demonstrated the technical feasibility of transvenous pacemaker implantation in small infants and shown favorable short-term outcomes [25]. However, for infants, because of their smaller blood vessels and less subcutaneous fat, larger pulse generators or two leads may not be accommodated. During the rapid growth phase in infants, there exists a significant discrepancy between the size of pacemaker devices and the body size of the patients. As transvenous leads pose an elevated risk of complications, such as vascular occlusion, thrombosis, and atrioventricular valve regurgitation, within this specific age group, epicardial pacing is considered the preferred method [25]. To minimize the risk of venous thrombosis in endocardial pacing systems, especially in patients weighing less than 15 kg, or when using single-chamber pacemakers, epicardial installation of the pacemaker system is preferred [24]. Consequently, based on the findings from previous studies, it has been proposed that endocardial pacing should be considered for patients weighing more than 15 kg, while epicardial pacing is recommended for patients weighing less than 10 kg. Furthermore, postoperative anticoagulation therapy for 6 months is advised for these patients [26]. In the present study, because of the patient’s young age and low weight, endocardial implantation was difficult. Therefore, epicardial implantation was chosen for all the patients. In the selection between single-chamber and dual-chamber pacemakers, research indicates that dual-chamber pacemakers can achieve favorable long-term outcomes and are also suitable for newborns and infants [27, 28]. Moreover, epicardial dual-chamber pacemakers have lower age and weight requirements and can fix electrodes in the right atrium-right ventricle or right atrium-left ventricle, thus reducing the incidence of heart failure [29–31]. The American College of Cardiology/American Heart Association guidelines recommends dual-chamber rate-responsive (DDDR) pacing rather than VVIR pacing [22]. For newborns and infants, it is important to choose a pacemaker that is lightweight, small, and with a long lifespan. In this study, single-chamber pacemakers and dual-chamber pacemakers were implanted in one premature infant and three cases, respectively. The smallest case was a newborn weighing 2.53 kg at 2 days old, who successfully received a dual-chamber pacemaker without any complications. In summary, application of dual-chamber pacing is a feasible approach to improve hemodynamic status and addressing ventricular deterioration caused by single-chamber pacing. However, there are fewer reports to support the use of this approach and further research is needed to determine the best implantation mode, develop devices with longer battery life, and prevent unnecessary pacing to reduce long-term morbidity rates [4, 19, 32]. In conclusion, based on other observations in this study, we infer that implantation of an epicardial pacemaker may be a safe and efficient strategy in neonates and infants, especially in the context of challenges related to venous access and the unfeasibility of receiving endocardial pacing.

Pacemaker treatment is associated with several limitations and potential risks. Some of the risk factors that contribute to occurrence of complications during pacemaker implantation include wound infection, endocarditis, inadvertent cardiac perforation, and superior vena cava thrombosis [20]. Permanent cardiac pacing continues to exhibit a high incidence of complications, even among adult populations [36]. However, the risk of complications is particularly pronounced in infants and children [37–40]. Among the most severe postoperative complications associated with CCAVB are skin damage and pocket infection, with the latter frequently attributed to inadequate aseptic techniques or pocket hematoma [21]. Studies have found that over 30% of infants with CCAVB and normal ventricular function before pacemaker implantation develop dilated cardiomyopathy in the early post-implantation period [33]. Another study has reported that 18% of patients encounter lead fracture and insulation failure, but advancements in technology offer potential solutions to address these challenges [19, 34].

## Conclusions

In summary, our data indicate that pacemaker implantation in newborns and infants is generally safe. Most children diagnosed with CCAVB before or after birth should undergo pacemaker implantation before adulthood, whereas those diagnosed with CCAVB *in utero* require earlier intervention [20]. Epicardial pacing is an effective and relatively safe method for treating CCAVB in infants, particularly in newborns and infants with this diagnosis. Early pacemaker implantation therapy is critical for these patients, provided that pacemaker indications are strictly controlled, appropriate pacing modes are selected, postoperative follow-up is performed, and pacemaker parameters are adjusted in a timely manner.

## Data Availability

All data generated or analyzed during this study are included in this published article.

## List of abbreviations

CCAVB: Congenital complete atrioventricular block
CHB: complete heart block
LQTS: long Q-T interval syndrome
DDDR: Dual-Chamber Rate-Responsive
DDD: Dual-Chamber Demand Pacing
VVIR: Ventricular Inhibited Pacing or Ventricular Demanded Pacing
